# The roots of metaphor: the essence of thought

**DOI:** 10.3389/fpsyg.2023.1197346

**Published:** 2023-08-01

**Authors:** Herbert L. Colston

**Affiliations:** University of Alberta, Edmonton, AB, Canada

**Keywords:** figurative language, metaphor, conceptual domains, cognitive duality, source domain, target domain

## Abstract

The essence of metaphor’s reliance on two domains, a source and a target, is argued as stemming from a fundamental characteristic of higher cognition—that of conceptualizing more than one cognitive/embodied domain at the same time. This cognitive duality is argued to underlie a plethora of conceptual activities including comparison, contrast, categorization, as well as metaphorizing. Why “two” domains seems the emergent and optimal means of such meta-cognition, rather than a higher number of domains, which might confer some advantages, is argued to arise from a grand compromise between an extreme necessity of humans to create and rely-upon shared complex meanings, and the complexities in enabling such shared meaning across multiple domains.

## Highlights

- Deconstructs metaphorical structures to demonstrate the profound possibilities of dual cognitive schematic activation.
- Argues for a deeper understanding of metaphor beyond mere source and target domains.
- Emphasizes that dual activation is not in itself sufficient for metaphor to occur.
- Points to a wealth of other similar situations where the presence of a duality, as in metaphor, can be a tipping point in the ensuing magnitude of complexity.

## Introduction

“Are not gross bodies and light convertible into one another, and may not bodies receive much of their activity from the particles of light which enter their composition?”

“Truth is ever to be found in the simplicity, and not in the multiplicity and confusion of things.”

— Isaac Newton,

Opticks, 1704

http://www.rarebookroom.org/Control/nwtopt/index.html

Metaphor has long been recognized as involving two things, frequently labeled domains—a *target domain* people are seeking to understand, and a *source domain* used to help with that understanding. Whether the connection between these domains allows for one-way or two-way traffic, is a matter of much discussion. And what those domains actually are, as some kind of conceptual structures or something else, is also a slippery issue (Gibbs, 2017). But *that* metaphor involves two of these things, is a well-received idea (although some accounts allow for a third *thing*, an emergent blend of targets and sources, Fauconnier and Turner, 2008).

Moreover, recent research has noted that most forms of figurative language, including metaphor, also invoke exactly two of these “domains,” albeit they do somewhat different things with them than metaphor (a smaller set of figures invokes only one domain, and one figure or at least figurativesque form—puns, can at times invoke more than two domains), (Colston, in press; Colston and Rasse, submitted). So it seems as if *two* is a magic number of some sort, at least in metaphor and figurative language (Colston, 2019). Why might this be the case?

The argument to-be presented in the present paper is that it is not so much that metaphor just *happens* to involve two domains. But rather that cognition itself, in a general sense, seems to involve representation and activation (whether in an information processing, an embodied cognition, or in some other sense), of domain-like things—activating them at times to do cognitive work (e.g., a predator animal coming upon the scent of a ground-dwelling prey, activating the domain of “food,” or “hunt,” or “vole,” etc., and acting accordingly). But once cognition moves from dealing with one domain at a time to two—and especially with both domains being active in some particular way at the same time, something very special is enabled. Having the neural machinery to hold one conceptual domain in mind, while invoking a second one, allows for a wide variety of cognitive, or perhaps meta-cognitive processes, including metaphorical thought.^1^ Animals equipped with such neural machinery can *compare* the two things (which is bigger?), *contrast* them (which is better?), *substitute* them (a long stick STANDS FOR a long arm), as well as *metaphorize* the two domains (positive affect IS warm sunshine). So such an ability seems to bridge the very earliest possibilities in thought about more than one thing at a time, leading all the way up to the most profound and meaningful multimodal poetic metaphors. The latter, also enable realization of similarities between two things (roses and love, sunshine and happiness, etc.), yet also open up a universe of meaningful possibilities (Rasse, 2022).

So the metaphorical move, going from one concept to two, is actually a move from a single conceptual domain into a seeming infinity of understanding, much the same way that a point in a geometrical sense, is singular and infinitesimally small, yet two points together define an infinitely long line. Metaphor thus seems to reside where advanced cognition begins, but it does not seem to have any reasonable end.^2^

### The power of two in cognition

As a means of demonstrating the usefulness of this notion of “two” in cognition, please consider the following brief anecdote:

When in middle school in the United States in the 1980s, the author was invited to partake in a class exercise, one commonly used in North American middle schools at the time, to make a point. The students in the class were invited to each select a lemon from a large bowl, to then study the lemon for a moment, and to then place it into a large covered basket. The returned lemons were then all arrayed randomly on a long table, and students were invited again, this time to search among the array of lemons to find the particular one they’d held before.

The task appeared ludicrous at first, how could we tell our lemon from all the others? Certainly the lemons weren’t identical, but their differences seemed utterly trivial—enough so to make the task seemingly fruitless. We were all surprised to learn, however, that though subtle, those minor differences among the lemons allowed us all to successfully and rather easily select which lemon was ours. And it required relatively little time to accomplish. We then made lemonade.

This exercise was intended as more of a social lesson that a perceptual and/or cognitive one. It was used at the time to demonstrate to a group of budding adults, that things which appear essentially alike on the surface, nonetheless have discernable differences that matter. After the exercise was over and we were drinking our lemonade, we were invited again to apply this lesson to ourselves and to our classmates, and by extension, to all people. The exercise was a means of allowing us to understand two seemingly contradictory things—firstly that people belong to a single category by virtue of having the characteristics that put us into that category, essentially our humanity. Which suggests that people are in general largely *alike* or very *similar* to one another. Yet at the same time, those same “unitary” people bear remarkable differences that afford the ability to identify individuals quite readily by their unique configurations of human characteristics, even within a large and similar crowd, suggesting extensive *differences* or *dissimilarities* among people.

In essence, people can seem very different in all of the capacities that make us human, yet we nonetheless by virtue *of* those very humane characteristics are also united into a single category. We are different, *and* we are similar. We are all unique, *and* yet we all belong to a unified category. All seemingly contradictory, yet also all true. It was a valuable exercise.

But what can this middle-school lesson about lemon’s and people’s similarities and differences tell us about metaphor and human cognition, or any presumed connection between the two? It turns out that the lesson reveals a fundamental underpinning of both higher human cognition and metaphor, and why you cannot have all of the former without the latter. In order for the middle school students in the example to succeed at their selection task, they must assemble and maintain two related but different things at the same time. They must use their sensory systems to assemble a percept of a particular lemon they hold in their hand, noting any distinguishing characteristics or features, discerning any patterns, etc., essentially forming a representation of that particular lemon in their mind. They must also have retained some form of similar representation from the original lemon they’d held earlier—to which they can now compare the live-held lemon. If the representations reasonably match, the student can determine that they have found their original lemon. If the representations reasonably mismatch, the student knows they have not found their original piece of fruit, and must continue searching.

This capacity to hold more than one representation in mind somehow *at the same time* (in the present case, one actively generated sensorily and another held in memory), is the very basis of the concept of *comparison.*^3^ Abundant theories are available to explain how these related but differing processes specifically take place, but their working together to afford comparison gives birth to a seeming infinity of conceptual possibilities including, *sameness*, *difference*, *resemblance* of varying strengths and sorts, *opposition*, *identicality*, along with various relations like *member-category*, *initial-secondary*, *cause-effect*, *concrete-abstract*, etc. One could thus argue that this ability to manage more than one representation at a time is the very birth of higher-order cognition (with the ability to form only individual solo representations serving as a precursor). And, as this article will argue, it also seems to be the birthplace of metaphor.

### How else is cognition done?

Before arguing that higher-order cognition based on cognitive parallelism is the basis of metaphor, lets first consider briefly how cognition operates in other ways, at least in generalities. As a starting point, let us consider the notion of *representation*. Representation has been a dominant metaphor for thinking about cognition for millennia, if not longer.^4^ The idea that the brain/mind somehow holds, has, maintains, makes, and uses ideas, thoughts, notions, or representations of various sorts, of things in our conceivable existence (and even outside of that existence), is an old one. In more modern cognitive science, we also have various notions about how things, be they concrete and material or more abstract concepts or ideas, are represented in the mind. These take many forms including recurring patterns of neural activation related to concepts, schemata, scripts, frameworks, domains, embodied simulations, mentalese, nodes in semantic networks, and many others.

There are also approaches that attempt to avoid the concept of “representation” altogether, instead arguing for emergent patterns of semi-chaotic neural activity in the form of dynamical systems, and others (Gibbs and Colston, 2012). But even these views hold that those dynamic activities are not completely random, but rather pattern according to things encountered outside the neural system, *by* the neural system, perhaps sometimes as a cascade from other neural patterns themselves, but also responsive in semi-regular ways to things external to the system. All told, our entire mental/experiential capacity seems to ebb and flow in accordance with our environment (i.e., we experience hunger when we need nutrients, we feel fear at a charging animal, we think “magpie” when we visually or auditorially encounter a particular bird species, or “unfair,” in response to a particular judgment about something).

We also seem to build complexity and nuance into this general idea of representation in the form of broader cognitive structures. So we think about component parts that make up larger wholes (e.g., the eraser, metal band, wooden shaft, encased graphite core, pointed end, etc., of a pencil). We think extensively about categorization, always struggling over whether a given thing belongs to this or that category (e.g., is a pencil a tool, a utensil, an instrument, a household-or workplace-or school-or other type of item?). We often connect categories in the form of modal, or tree-like, or nested, or other kinds of networks (e.g., mammoths were a branch of proboscideans that went extinct, the U.S. Air Force used to be part of the U.S. Army, but was then made separate after the second world war, etc.).

We also increasingly must cobble together new representations for things that appear or are invented in our environment. So we have “solar shingles” for roofing materials that can collect and transfer solar energy, or “fobs” which originated as straps to hold pocket watches but now refer to electronic remote-control keys for locking/unlocking things, etc. And of course many of these are based on resemblances of many kinds with pre-existing, pre-represented things (e.g., a computer mouse).

From these sorts of resemblance-based new representations we can begin to talk about analogies. Or, a sort of representation where the form, pattern, or function/structure of one representation is extended to that of another thing. So we might say that a car fob is the modern analog of a car key. Or we might say the CPU of a computer is analogous to the brain of an animal, or the headquarters of a complex organization. This is all not so much an act of constructing a representation from scratch, but rather in a *transferring* of something from a representation of one thing onto another.

And finally from there we can morph into the full-on leap-of-faith of the world of metaphor where representations do not involve things just being analogous to other things, but rather when things are purported to *be* those other things. So we drop the scaffolding or pretense around a *comparison* or *resemblance* (functionally or otherwise) of A to B, and just state, show, act, think, etc., that A *is* a B. The particular means of this metaphorizing between two things varies of course across language (as well as languages), image, sound or any other way we can encounter/sense things, but they all posit the relationships between A and B quite boldly, in a way imposing B-ness onto A.^5^^,^^6^

Indeed, one might *demonstrate* some of the very representation-building processes *discussed* above in our very attempt to understand what metaphor is. We might argue for instance that metaphor is a bit of categorization grafted onto analogy—we apply the structure or function of B to that of A (analogy), by saying that A is a member of the category B (categorization). Indeed, this seems the very approach adopted by the Categorization account of metaphor (Glucksberg, 2001).

But even in all of the above ways in which we make and have representations, we are still always hovering around the notion or quantity of “twoness.” We build representations in our minds/bodies (1) of things in our environment (2)—with, as a caveat, our minds/bodies being fair game as part of that environment. Or we represent the entirety of a thing (1) as made up of component parts (2). We also make dichotomous decisions often as to whether a given representation (1) belongs or does not belong to a given category (2).^7^ When we assemble categories into broader structures we are often doing so via a bit of metaphorizing, as when we apply the shape of a tree (1) to an assemblage of categories of animals (2), or when we borrow the structure of an actual mesh net, as in a fishing net (1), and apply it to a an array of categories of things, as in a semantic network (2). Our constructions of new representations of new things (1) as mentioned, usually borrow from pre-existing representations of older things (2), as in a computer port, borrowed from the idea of a shipping port. Analogies *borrow* a structure of one domain (1) and apply it to another (2), as in, rainforests are the lungs of the planet. And metaphors *impose* something from source domains (1) onto target domains (2).^8^

Of course one might simply view all of these related ways in which we assemble representations as involving “two” or twoness, as simply what results once we expand beyond the singularity of just having a single thing—it might be seen as just the inevitable result of the building of complexity. You begin with nothing. Then you have something. Then for any degree of differentiation to be achieved, that something must be discerned into different somethings, so we move from having just one thing, to having another thing, such that we end up, at least at first, with “two.”

But there are a huge number of ways in which our complex worlds and our complex minds operate with many levels greater than two. Just going back to the notion of wholes and parts, we are very comfortable with the idea of wholes requiring large numbers of parts. We also readily operate with a plethora of differing categories, and categories with many members. So we have the category of vehicles as well as lots of kinds of vehicles. Among the category of two-wheeled vehicles we have bicycles, motorcycles, pull-carts, push-carts, chariots, trailers, rickshaws, etc.

But once we move to the level of analogies or metaphor, we seem constrained more by this notion of twoness. We very typically talk about analogies in terms of targets (1) and vehicles (2), (e.g., final editing of a paper is like a final polishing a shiny surface—careful vigilant actions to remove all the remaining imperfections [i.e., “smudges” or “typos”]). And metaphors as mentioned at the beginning of this article involve source (1) and target (2) domains.

So perhaps there is just something germane to metaphors and analogies that likes the use of only two domains. But the question remains as to what this “something” actually *is*. Are there reasons that we do not usually go beyond two domains—something about three or more that is detrimental? Or is the meaningful move from dealing with just one thing to a pair of things all that can be achieved? Or is there some characteristic of two domains that emerges as being most beneficial. Or is some combination of these possibilities the answer? To further our understanding of this issue, let us move away from metaphor for a moment and briefly consider other forms of figurativity or figurativesqueness that also make use of domains.

### Other implicit invocations of domains in figurativity/figurativesqueness

Metaphor is not the only way that we use domains in our creations of representations. We also craft metaphors both linguistically and within many other kinds of media. But we’ll confine the discussion here to forms of figurativity/figurativesqueness that are conducted via language.

Colston (in press) and Colston and Rasse (submitted), provided an analysis and deconstruction of 15 types of figurative language, or at least figurativesque language.^9^ The forms discussed were; *rhetorical question, asyndeton, metonymy, hyperbole, tautology, antimetabole, metaphor, verbal irony, idioms, proverbs, simile, oxymoron, onomatopoeia, allegory,* and *puns*. This deconstruction involved looking at the generic structure of the figurative/figurativesque forms and determining, (1) how many domains were invoked, (2) what those domains were, (3) what is done with those domains, and (4) what doing things with those domains accomplishes for interlocutors using the figurative/figurativesque forms.

**Figure 1:**
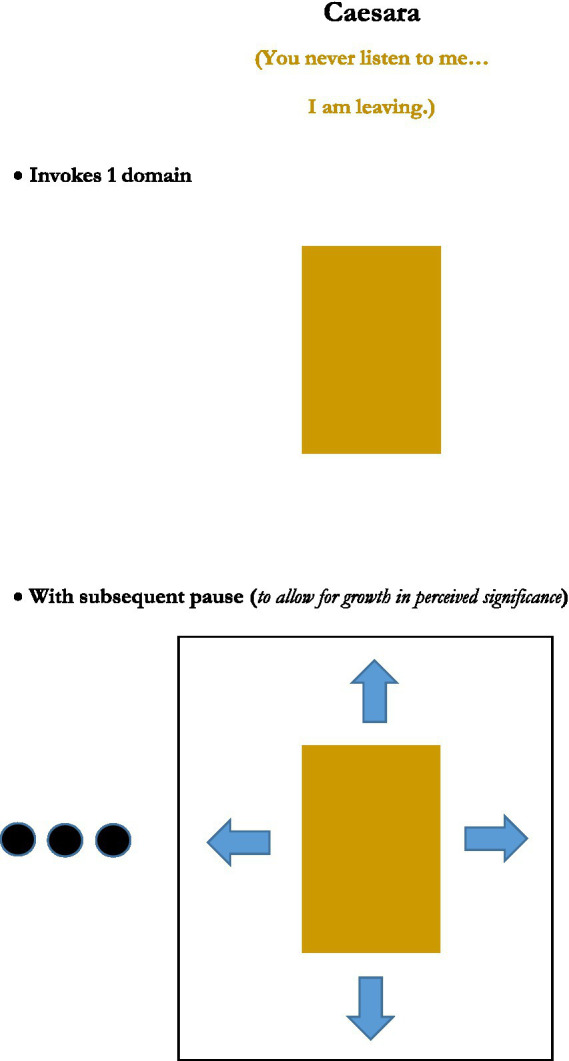
Caesara.

**Figure 2:**
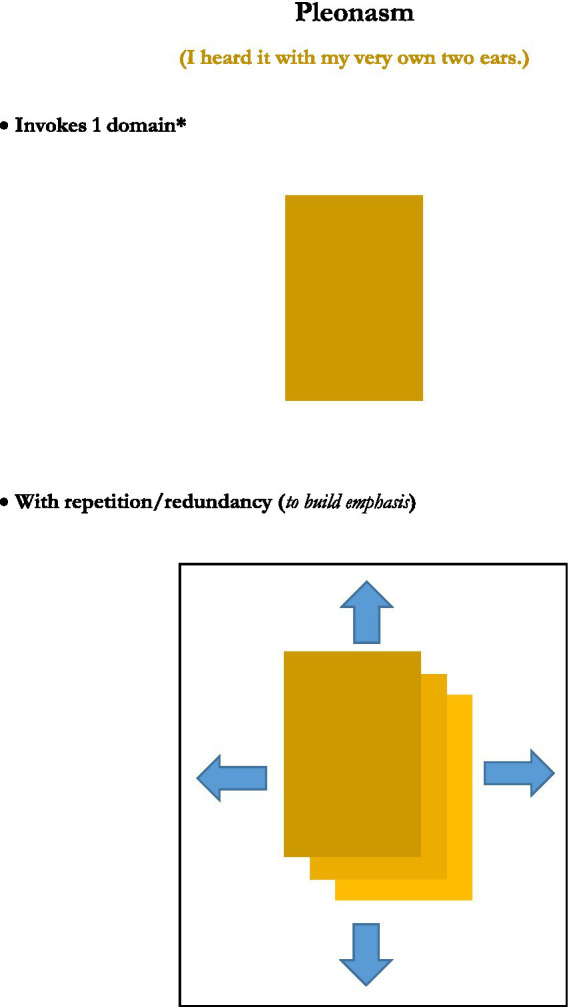
Pleonasm. *Might be considered ‘two-ish’ domains.

**Figure 3:**
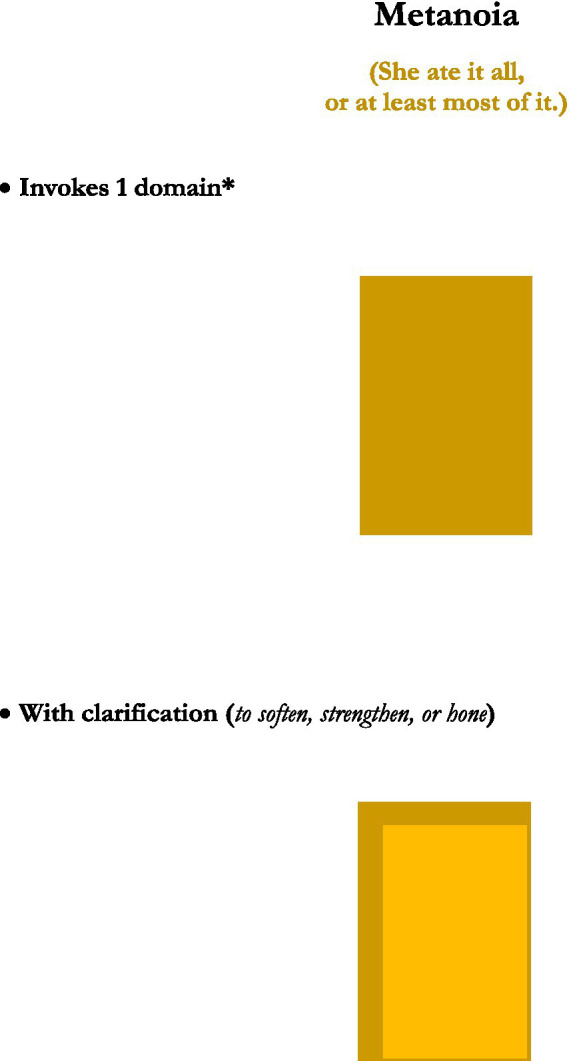
Metanoia. *Might be considered ‘two-ish’ domains.

**Figure 4:**
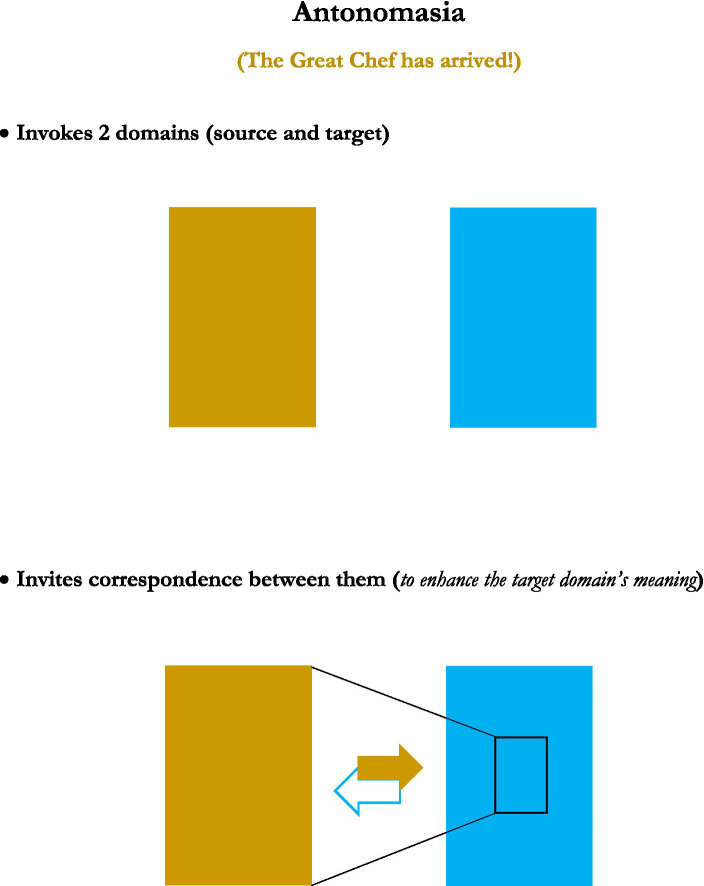
Antonomasia.

**Figure 5:**
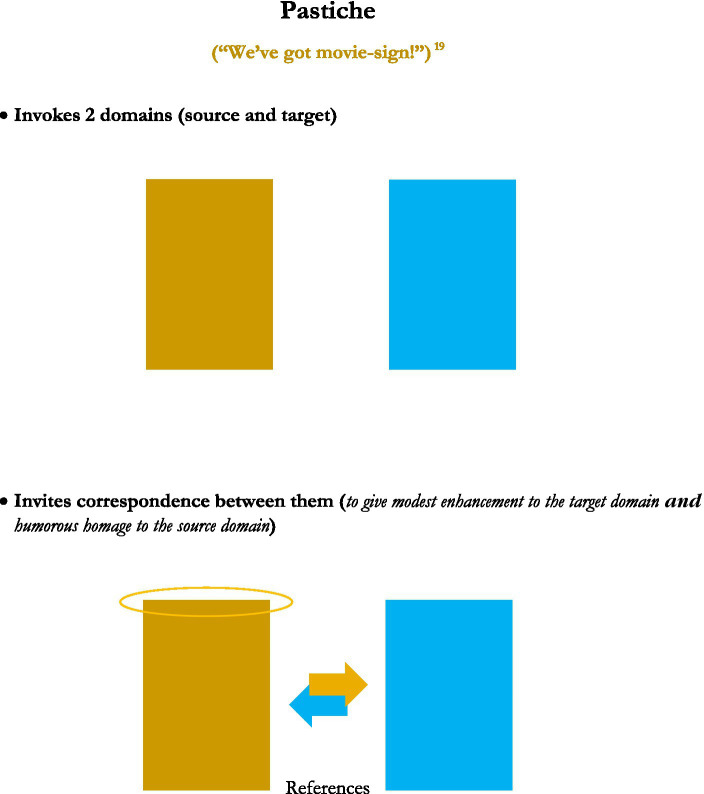
Pastiche.

This analysis was achieved in part by consideration of generic depictions of the structures of the figures, presented in graphical images. Rather than reprint the set of graphics of those figures’ structures here, five new graphics, for the figures or figuresque forms of *caesura, pleonasm, metanoia, antonomasia*, and *pastiche*, are presented instead (see Figures 1–5). These may serve as further examples of the range of figure/figuresque types discussed in Colston (in press) and Colston and Rasse (submitted). They can also represent the primary outcomes of those analyses. The upshot of that analysis is it revealed and afforded new insights into, why we have the range of figures we do, why some figures are more prominent than others, and why a very few of those figures seem to be the most prominent in terms of frequency of appearance and usage (e.g., metaphor, irony and perhaps metonymy).^10^

The five particular figurative/figurativesque forms presented here were selected to demonstrate one of the main emergent characteristics of the analysis provided in Colston (in press) and Colston and Rasse (submitted) on the 15 original figures—that most figures invoke only one or two domains^11^, but that some types might invoke “2-ish” domains in that only one actual domain is being used, but it is repeated, or used but then inverted, or in some other way invoked more than just one time. But the main point is that the majority of figures invoke two domains, just like metaphor and analogy do.

#### Caesara

This figurativesque form is not so much an actual linguistic construction, excepting in the form of punctuation occasionally used to indicate its usage in text^12^, but it nonetheless involves a delivery characteristic in speech that behaves like figurativity. This characteristic can have a figurative effect of sorts on the comprehension of the domain being invoked—lending it a degree of seriousness, profundity, weightiness or significance. Caesara is effectively the same idea as a dramatic pause—holding the speech stream for a moment after a domain is invoked by an utterance, to allow lengthier consideration of the invoked domain, which can frame it in a more pronounced way. This profundity might ensue by allowing time for whatever embodied simulations the utterance might conjure to blossom, or to invite greater incorporation of the invoked domain within its background context (depending on one’s preferred processing model). But either way, it ultimately makes the domain from the preceding utterance more heady in the perception of the interlocutors.

#### Pleonasm

Pleonasm also invokes only a single domain, but here pleonasm is considered a “2-ish” figurativesque form in that it invokes that domain more than one time. Its use of restatement, redundancy, or other similar forms of repetition, as in (“I cannot believe I ate the whole, entire, complete thing!”), where the same essential domain, in the case of the example, the eating of the *entirety of something*, is repeated for emphasis. The repetition, in the example of varying terms for “entirety,” seems to emphasize or especially highlight that particular characteristic of the invoked domain, the entirety of a thing eaten. Otherwise the redundancy seems unnecessary since one such term should suffice to convey the essential meaning.

#### Metanoia

Metanoia is another “two-ish” figure (or figuresque form), in that it also invokes only a single domain, but metanoia then re-invokes the domain a second time, immediately after the first, with greater specificity. For instance, a speaker might say, “this is a form of figurative language, or at least figurativesque language” to first orient the hearer/reader to the general domain being invoked, perhaps because that general domain is more recognizable or familiar. But then the speaker qualifies the referenced domain in a subsequent adjacent construction that softens, strengthens, or somehow hones the referent domain with greater precision. Such a technique might simply reflect how people talk in normal causal conversation, where an initial idea comes to mind at first, is then uttered, but then is qualified upon further contemplation. But it could also serve to demonstrate the speakers recognition of the resemblance of the more specific description with the initial broader one, to note the subtle distinction between the two, but to not give the distinction great credence. All in all it seems to suggest the broader description is generally apt, yet to also acknowledge subtle details at play.

#### Antonomasia

And finally we have two examples of more genuinely 2-domain figures, which each operate a lot like metaphor in that they invoke two separate domains and invite correspondences between them. But each figure differs slightly in the nuance of those invited correspondences. For antonomasia, as in saying, “Hey, it’s the Formula One Racer!” to refer to a person who had recently exhibited great driving skills in chauffeuring several passengers up a windy mountain road, a speaker is invoking two domains. One is the actual referent, a person/driver in this case. The other, the source domain, is a member of a class of individuals known for some exceptional characteristic—Formula One Racers, who are distinguished by superior driving abilities.

Antonomasia feels a lot like a metaphor—using a source domain (Formula One Racers) to highlight something about a target domain (an individual person’s driving ability). But it seems also a little different as a cross-domain mapping in that the individual in question already has something from the source domain—driving skills. The mapping in this case thus concerns the *quality* of those skills, not so much their mere presence. Other metaphors seem more involved in imposing characteristics from a source domain onto a target domain that are less-obviously already present. So antonomasia seems to have some components of hyperbole in its operation, in inflating a characteristic of a domain to draw attention to it (Colston, 2019). Or even that of metonymy, in that a characteristic of a target is used to refer to that target in its entirety, in a seeming form of substitution. So antonomasia resembles metaphor, but has interesting hints of hyperbole and metonymy present as well, possibly allowing for its differing designation.

#### Pastiche

And lastly we have pastiche, which also resembles metaphor in the use of a source and target domain. Although pastiche seems a bit like allegory in that it can apply quite broadly and often is not contained only in a particular linguistic utterance or other smallish meaningful construction. For allegory, that broad application is usually for some lesson, moral or other characteristic from a source domain, to apply to a relatively open class of target domains in the world of human events (e.g., “The Boy who Cried Wolf” can apply as a lesson to many human situations). For pastiche, though, the broadness lies more with what can be shared or similar between the source and target domains. And with pastiche the direction of meaning enhancement seems more nuanced. An instance of pastiche usually draws correspondences between a source and target domain, but often more for an honorary nod, or respectful, albeit often humorous, acknowledgement of the source domain from which a characteristic originally resided. But it can also lend a boost of understanding to the target domain, as is usually the main function of metaphor.

For instance, in the classic American television/internet comedy shows Mystery Science Theater 3,000, and RiffTrax, among many, many others, some aspect of a particular target situation, will often be highlighted with some characteristic from a different source domain situation. A comment from an instance of riffing (comedic commentary about some scene in a movie, usually one of poor quality), or a skit involving play-acting with some motif or theme from that movie, can thus borrow content from a source domain, applying it to the target domain. So if a movie scene involves a chase where one car rapidly leaves another car behind, a commentator might mimic the classic “Beep, Beep” call of the Road Runner character from classic American Warner Brothers cartoons in the mid 1900s. The mimicry both highlights a characteristic of the target domain (one thing soundly defeating another thing in terms of their relative speed of motion), by tying it to a well-known cultural meme from another source (the Road Runner cartoons) where that characteristic is caricatured. But the mimicry also gives homage to that source domain content itself.

Pastiche resembles parody in that the latter often uses a source domain content or framework to belittle (humorously or not) a target domain, occasionally making fun of both the target and source domains in the process. But pastiche is more honorary in its use of a source domain to lend a humorous take on a target domain. For instance, many musicians will uses instances of melody in a song to give both a light-hearted lilt to the composition being performed, yet tip-their-hat in an honorary nod to the source domain from where the content was borrowed.

In these five cases (bolded in Table 1), as in the 15 reviewed in Colston (in press) and Colston and Rasse (submitted), we have the same emergent findings. Most domains use one or two domains, but the majority use two, or at least two-ish. Only puns, which typically involve two domains, can on occasion use more than two domains:

We can see that most of the Table 1 figures/figurativesque forms involve using two domains to invite correspondences between them, in subtly differing ways (e.g., *metaphor, idioms, proverbs, simile, allegory, antonomasia*, and *pastiche*). A smaller group use two domains more for contrast, nullification, substitution, and the like (e.g., *verbal irony, oxymora, metonymy*).

**Table 1:** Figurative/figurativesque forms and their numbers of domains.

One domain	Two-ish domains	Two domains	Two-plus domains
Rhetorical questions	Metonymy	Metaphor	Puns
Asyndeton	Hyperbole	Verbal irony	
**Caesara**	Tautology	Idiom	
	Antimetabole	Proverb	
	**Pleonasm**	Simile	
	**Metanoia**	Oxymoron	
		Onomotopeia	
		Allegory	
		**Antonomasia**	
		**Pastiche**	

We see also that the figures invoke these domains and do fairly straightforward things with them. Of the five figures reviewed here, they inflate the domains in a way to increase their importance (*caesura* and *pleonasm*), they use a domain in a general sense first to draw attention to it, but then to clarify its invocation (*metanoia*), or as mentioned they draw correspondences between two domains (*antonomasia* and *pastiche*).

And all of this domain invocation and manipulation ultimately gives rise to pragmatic effects and figurative meaning nuance. And all of this is in the service of enabling the construction of mental representations—of meaning-making. Which brings us back to the question of why the invocation of two or two-ish domains, which seems predominant in this form of meaning-making. We return to this question now.

### What is significant about *two*?

There are three parts to the argument attempting to explain the significance of “two” in this form of meaning-making. The first involves the quintessential human characteristic of a need-for-meaning, with an emphasis on the word “need.” The second is the importance of recognizing that a need-for-meaning is fundamentally a need-for-*shared*-meaning. The shared or social aspect of meaning-making, by figurativity and metaphor and all the rest, is key to understanding the importance of “two.” And the third part of the argument addresses the specific question about what makes “two” seem special. It turns out it may all involve a grand compromise.

#### The need for meaning

Part of what makes “two” special is not so much a wondrous quality of twoness *per se*—some exceptional quality about “two” that sets it apart and above any other technique of, or blueprint for, meaning-making. Rather, the utility of “two” arises from how it so neatly fills a particularly strong *need* in people—that of supplying meaning.

People do not just use pairs of domains to craft new representations because we can, or because doing so is nifty, or interestingly creative, etc. (although it can be). Rather, we use pairs of domains to craft new representations because we *must*. Humans and human minds have gone out onto a limb, evolutionarily, in that we have committed our survival to our ability to use our minds and bodies to make ever-growing sense of the world and to then act on those understandings. Now all animals and other life forms with sensory systems to survey their environments, and action-plans derived from DNA or learning systems, make use of such “meaning” (i.e., stored or derived representations, of a sort, of the world). Sun-loving plants will grow toward the sun, but not toward shade. Animals will engage in stalking behavior when they have detected prey, but not when they have not. Squirrels will store nuts when the temperature drops, but not before, etc. And these actions which are necessary for survival, as said, are encased in a way within the lifeforms via DNA-encoding or through learning and retention by nervous systems from observation or experience (Colston, 2019).^13^

But these forms of useful “meaning” are relatively simple in comparison to what humans do. Other lifeforms can rely on their DNA endowment and modest learning to survive. But we are much more dependent on *extensive* learning—on acquiring, developing, maintaining and expanding our learned representations of the world for our survival. An average modern person spends years of their lives in focused study and learning of skills, trades, abilities, knowledge-sets, expertise, etc., to function in the world. And increasingly, we must continue that learning throughout our lifespans since the knowledge required to maintain our survival changes and expands rapidly.

But even all this carefully crafted and retained knowledge is not enough to ensure our survival. We also must *share* that knowledge with others, and them with us. Our collectively shared knowledge, culture if you will, is our primary means of survival. And language, it can be argued, is the primary means by which all this knowledge-sharing takes place.

So humans have evolutionarily committed themselves to deriving, creating, building, sharing and using collective “meaning,” in order to survive. This need for understanding puts enormous pressure in us to get and to have meaning. We are uncomfortable if we do not have it. We strive to achieve it. We clamor to have more of it. We are devastated if it is taken from us. We are extremely happy to achieve it. We like it when it is fresh, new, more encompassing. We build entire cultures, institutions, societies, etc., around it, etc. This meaning/understanding involves the “anatomy” of our worlds (its component parts and their arrangements). These often take the form of parts, associations, relations, connections, comparisons, categorizations, hierarchical and network arrangements, etc. And meaning also involves getting the “physiology” of the world to understand how all that anatomy works together to function. All of this requires meta-representational thinking in the form of two things together making a third thing, or two things with another thing between, across, among, shared by, them, etc. And one major form that this understanding takes is the understanding of one thing, in terms of another thing—the birth of metaphor.

So all told, we greatly need meaning, metaphorical and other kinds, causing us to create and share/borrow it extensively, and of finding means of expanding it however we can onto the myriad of things that remain unknown. So metaphor is not merely a clever way to say something. Nor is it just a structure of sorts in the mind/body.^14^ *It is more fulsomely a fundamental human individual and social requirement, one key part in our desperate attempt to fulfill our need for meaning, upon which our survival depends.*

#### The need for *shared* meaning

Part of what makes “two” useful for building representations to fulfill our need for meaning is the fluency with which *meaning-built-from-“two”* can be shared. For something like metaphorical meaning to bridge two people means that the people must already share something about the individual component parts of the metaphor (i.e., the source and target domains). If, for example, the conceptual metaphor, KNOWLEDGE is FOOD is invoked in the linguistic metaphor, “they ate it up” in response to a question about how an audience received a new idea, people must already have some individual grasp of both knowledge and food. One could argue that this explains why so many metaphors are based on either embodiment or culture (or both), in that a pair of interlocutors, in the case of successful metaphor understanding, are likely to share those things. They would share similar embodied experiences by virtue of having a similar human mind and body. And they would share a culture by virtue of overlapping on a set of beliefs, understandings, values, etc. So “two” works well on this point since it aids, expands, and shares something pre-existing in pairs, or more, of people. Yet it does not reach too far. Should a representation demand shared understanding of three or more domains, then the likelihood of adequate sharedness would diminish—the more things people must share for understanding, the less likely understanding will be. Metaphor, as such, quits while it is ahead.

A related advantage of building representations out of two domains is that the potential meaning can be pretty far-reaching. Conceptualizing something *positive* from a human’s experience, in terms of physical *upwardness*, can encompass an enormity of content. We have a lot of things we might consider positive experiences. And we have a myriad of ways of referring to upwardness. Each of these domains can have a lot of variability within themselves [e.g., a skyscraper and a cloud have a lot of differences (e.g., one is a human-built material structure, the other is a gaseous weather phenomenon, etc.)]. But they share, *upwardness*. The same holds for positive experiences (e.g., feelings of relief and orgasms—one can come from crossing items off a to-do list and the other is a raw sexual phenomenon). But a metaphor that uses one thing in common from one domain (positive polarity) in connection with one thing shared by the other domain (upwardness), aligns all those things together in a common relationship (e.g., good things are up). This is a lot of meaningfulness lifted with a very small lever.

And finally, the nature of a metaphorical linkage between two domains (e.g., knowledge IS food), being based a claim of on *oneness*, or *being the same thing*, might be particularly useful in achieving shared meaning. Were a speaker to posit a mere *resemblance* or *modest overlap* between two disparate things (e.g., saying, “a piano is like a bird”), they invite a consideration of what the things have in common (e.g., making musical sounds). But the comparison allows for a lot of variability to remain (e.g., birds are alive and pianos are not), which can itself vary across comprehenders (e.g., another person thinks birds are light and pianos are heavy). The typical metaphorical construction, however, pressures comprehenders to more thoroughly align on the relationship between the two things, emphasizing and enhancing that sharedness—both the sharedness between the aligned domains and that between the interlocutors.

#### The grand compromise

Related to the above ideas about metaphor expanding and enriching meaning but not going too far (e.g., “quitting while it is ahead”)—leveraging a connection between two things likely already possessed by interlocutors, and that leveraging being powerful (aligning a lot of content), yet limited, is the idea of a grand compromise. Metaphor, being built on two domains, might land upon the ideal degree of meaning-making. It might maximize the meaning shared between people but also minimize any hindrances in achieving that shared meaning.

Colston (2019), illustrated this compromise through a comparison with sexual reproduction in biology. Species need to change in order to adapt to changing environments. Mixing genetic material from different individuals in reproduction, aids in this diversification—an offspring of two different individuals will differ somewhat from each of those parents. But as a logical conclusion, why did not reproduction in general^15^ then combine genetic material from more than two parents? Why not three or more parents, which might increase an offspring’s fit with an environment by sampling and averaging from a greater pool of individuals (akin to better representation being found with larger sample sizes, in different kinds of research)? One reason might simply be the advantage of coordination of reproduction when only two parties are involved, versus more than two. Anyone who has tried to plan a meeting time with busy people likely understands this issue—it is much easier to coordinate with just two attendees, versus more than two. So the need for genetic variability over time might be best met all around by the compromise of combining genetic material from two individual parents rather than three or more. Maximizing diversification, while minimizing coordination complexity and difficulty. “Two” is where those constraints seem to be met optimally^16^.

A similar situation might hold for *expanding* meaning possibilities, while also ensuring the *sharedness* of any new meaning representations. Understanding one domain in terms of a second domain, might enable a nuanced or altogether novel understanding of the target domain, while still guaranteeing that the new understanding is within the shared reach of different individuals. Meaning expansion coupled with meaning sharedness. “Two,” again appears to be a useful compromise.

## Conclusion

The fact that metaphor is built on the primary idea of one thing being structured in terms of another thing, all for the purposes of creating new meaningful mental representations, was argued to have a degree of special import, resting on the notion of “two” as the number of domains used. A sizeable set of similar figurative and figurativesque forms was reviewed to show their similar gravitation toward the usage of two domains. Most of those forms use two domains or something very similar to two domains, in their differing yet comparable processes for leveraging new meaning and pragmatic effects.

An argument was also presented that the usage of two domains for meaning-making stems ultimately from a major early-cognitive step of meta-representation where more than one conceptual domain can be considered at a time. Such a step enables comparison, categorization, and ultimately metaphoricity.^17^

The need for metaphorical representations to be shared by interlocutors, arising from a baser need-for-meaning, endemic of the human evolutionary trend on relying on mental representations for survival, was also emphasized. The reliance of two domains for such shared meaning was presented as arising from both the advantages of what pairs of domains enables, as well as challenges if the number of domains for meaning-making expands beyond two.

And finally, this tendency toward “two” for meaning-making was presented as resembling the biological compromise of relying largely on two sources of genetic material for reproduction—optimizing the diversification of individuals in response to adaptation pressures, with the realities of reproductive coordination.

Our understanding of metaphor began as a tool for meaning-enhancement in language. It then moved to serving as a predominant way in which we mentally organize cognitive and embodied experiences, in language and beyond. Now it is being seen as a fundamental characteristic of shared cognitive/embodied meaning-making necessitated by a species that is extremely dependent on assembled meaningfulness for survival—to predict, navigate, and increasingly to manipulate its environment. This has been a lengthy progression.

A subtle comparison was made earlier between nervous systems based on neurons versus ones based on fungal threads or hyphae as used by mycelia. Such a comparison might also hold insights for the history and evolution of thinking about metaphor. Our original idea was that metaphors were essentially interesting “fruits” found only in language. This might be akin to our older thinking that mushrooms and other fungi were also simply a form of plants with roots that fed on dying plant matter. We now know that mushrooms are merely the flowers of a much more extensive and extremely complex and arguably “intelligent” lifeform known as mycelia, warranting its own taxonomic category alongside plants and animals, and more closely resembling the latter. And also being necessary for the very existence of many other forms of life. So, as we once underestimated mushrooms, we may have also underestimated metaphors—they were once relegated to fanciful language forms, then upgraded to having prominent presence in everyday speech. They have since been understood to reside in all forms of human thought and creative activity. Now they might be realized as fundamental to the very earliest bases of representational cognition.^18^

## Data availability statement

The original contributions presented in the study are included in the article/supplementary material, further inquiries can be directed to the corresponding author.

## Author contributions

The author confirms being the sole contributor of this work and has approved it for publication.

## Conflict of interest

The author declares that the research was conducted in the absence of any commercial or financial relationships that could be construed as a potential conflict of interest.

## Publisher’s note

All claims expressed in this article are solely those of the authors and do not necessarily represent those of their affiliated organizations, or those of the publisher, the editors and the reviewers. Any product that may be evaluated in this article, or claim that may be made by its manufacturer, is not guaranteed or endorsed by the publisher.

## Footnotes

^1^The psychological concept of “association,” as in associative learning, may be considered a parallel, analog, or possibly a precursor to a dual consideration (where a neural system “learns” that two things go together [e.g. sun and warmth]). But in metaphorical cognition, the association concerns broader cross-domain resemblances or similarities, rather than just mere co-occurrences or other relations (e.g., causal).

^2^This is not to argue that metaphor opens up a genuine and literal infinity of possible meaning, who are we to know what such a thing even is? But the extent of metaphorical meaning does seem to have a fair degree of astounding open-endedness, in that we have certainly not reached an end to the extent of metaphorical meaning/understanding, nor does one seem in sight.

^3^Of course comparison can also take place between two sensorily-active representations, as in comparing two things within sensory access at the same time (which would still likely involve some memory components albeit ones of shorter storage durations), and it can involve two representations derived essentially from memory. And comparison needn’t be held to just two things. But a necessary and sufficient condition of comparison seems to be that two things are held at the same time somehow as representations in a nervous or other comparable cognitive system.

^4^Indeed, one could argue that representation as a process might have historically and evolutionarily preceded humans’ representation of “representation” as an entity. Although one wonders what a non-human member of a highly-social animal group experiences when an alpha-member leads a protest against a potential intruder—whether or not a vague notion is in place of another individual representing “me,” or the group.

^5^Or of course, blending A and B together to a degree and then imposing that emergent new thing that is both A and B and more, onto A (with maybe some spillage also onto B) as conceptual integration would argue.

^6^Though the specific nature of this “B-ness” transferred to “A” is undergoing significant debate. Traditionally, it was the relative concreteness that seemed to predict when such a projection would occur. Other accounts have based transfer likelihood upon relative frequency (Winter and Srinivasan, 2022), or “simulatability” (Colston, 2019) of source versus target domains.

^7^And we are often uncomfortable if something does not neatly belong or not, or if something can belong to more than one category. We prefer the neatness of one or the other.

^8^With of course the additional nuance brought to this pairing, which varies across differing accounts of metaphor.

^9^Some figures were discussed in both publications, other figures appeared in one or the other publication, but the overall discussion encompassed the 15 unique forms mentioned here.

^10^“Frequency” must admittedly be based on speculation, for it is notoriously difficulty to determine the actual preponderance of different figures in the enormous and intractable population of human communication (Colston, 2019). But they do appear most prominent in terms of the attention given them by figurativity scholars.

^11^Puns were the one exception, invoking potentially more than two domains. But even puns usually invoke only two domains.

^12^The punctuation symbol || is used on occasion in written scripts and other textual forms of spoken language, to indicate the caesura or dramatic pause, as in, “To be, or not to be ||, that is the question” (Shakespeare, 1988).

^13^“Nervous” system not being restricted the neurons, but also possibly including fungal threads or hyphae as used by mycelia.

^14^Not to belittle the insight behind this realization, which arguably rejuvenated the modern understanding of metaphor (Lakoff and Johnson, 1980).

^15^Some such experimentation did indeed take place in biological history on Earth, as did tinkering with varying forms of symmetricity. But a great many animal lifeforms gravitated toward sexual reproduction, as well as bilateral symmetricity, at least in part (e.g., locomotion, sensory systems, etc.).

^16^A similar form of optimization might also be found with metaphor, and beyond simply that of the involvement of two primary entities. The *extent of semantic reach* across two domains invoked in metaphorical communication (and other uses of metaphor) also seems to strive toward a form of optimality. In this case, the compromise is between the expansion and novelty of meaning brought by larger reaches, and the comprehensibility concomitant with smaller ones (e.g., “optimal innovation,” Giora et al., 2004).

^17^The notion and utility of using two domains to create new metaphorical meaning is not necessarily new, it has been discussed in various guises regarding competing metaphor theories (e.g., Conceptual Metaphor versus Conceptual Integration). But the noting of this usage of “twoness” across an entire array of differing figures is a more novel development.

^18^“Representational,” again, is meant to encompass accounts based both on the explicit notion of “representativeness,” where the mind somehow represents something in the world (which of course, includes that mind) as well as accounts that attempt to do away with the metaphor of representation—basing cognition on some other processes of mind/world alignment. But in either case, the bridging of oneness to twoness, seems to be one of the essences of higher-order cognition.
